# Nondestructive monitoring storage quality of apples at different temperatures by near‐infrared transmittance spectroscopy

**DOI:** 10.1002/fsn3.1669

**Published:** 2020-05-27

**Authors:** Zhiming Guo, Mingming Wang, Ali Shujat, Jingzhu Wu, Hesham R. El‐Seedi, Jiyong Shi, Qin Ouyang, Quansheng Chen, Xiaobo Zou

**Affiliations:** ^1^ School of Food and Biological Engineering Jiangsu University Zhenjiang China; ^2^ Beijing Key Laboratory of Big Data Technology for Food Safety Beijing Technology and Business University Beijing China; ^3^ Division of Pharmacognosy Department of Medicinal Chemistry Uppsala University Uppsala Sweden

**Keywords:** apple storage quality, near‐infrared transmittance spectroscopy, partial least square, temperature compensation, variable selection

## Abstract

Apple is the most widely planted fruit in the world and is popular in consumers because of its rich nutritional value. In this study, the portable near‐infrared (NIR) transmittance spectroscopy coupled with temperature compensation and chemometric algorithms was applied to detect the storage quality of apples. The postharvest quality of apples including soluble solids content (SSC), vitamin C (VC), titratable acid (TA), and firmness was evaluated, and the portable spectrometer was used to obtain near‐infrared transmittance spectra of apples in the wavelength range of 590–1,200 nm. Mixed temperature compensation method (MTC) was used to reduce the influence of temperature on the models and to improve the adaptability of the models. Then, variable selection methods, such as uninformative variable elimination (UVE), competitive adaptive reweighted sampling (CARS), and successive projections algorithm (SPA), were developed to improve the performance of the models by determining characteristic variables and reducing redundancy. Comparing the full spectral models with the models established on variables selected by different variable selection methods, the CARS combined with partial least squares (PLS) showed the best performance with prediction correlation coefficient (*R*_p_) and residual predictive deviation (RPD) values of 0.9236, 2.604 for SSC; 0.8684, 2.002 for TA; 0.8922, 2.087 for VC; and 0.8207, 1.992 for firmness, respectively. Results showed that NIR transmittance spectroscopy was feasible to detect postharvest quality of apples during storage.

## Graphical abstract

Near‐infrared transmittance spectroscopy coupled with temperature compensation and chemometric algorithms was applied to detect the storage quality of apples. Results showed that near‐infrared transmittance spectroscopy was feasible to detect postharvest quality of apples during storage.

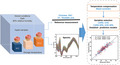

## 1 INTRODUCTION

Apple is one of the most popular fruits in the world, and it has a variety of bioactive ingredients, which are beneficial to human health, such as cellulose, vitamins, and minerals (Escribano, Biasi, Lerud, Slaughter, & Mitcham, 2017). Among them, soluble solids content (SSC), firmness, titratable acid (TA), and vitamin C (VC) are four important quality indicators of apples (Cortés, Blasco, Aleixos, Cubero, & Talens, 2019). SSC can reflect the sweetness of apples, firmness is related to apple tissue structure, and TA and VC are associated with the composition of the apple cells. These quality indicators have direct impacts on consumers' preferences and purchasing behavior (Cortés, Cubero, Blasco, Aleixos, & Talens, 2019). Therefore, the determination of above apple quality indexes is of great significance for meeting the needs of consumers.

Traditional apple quality detection methods are visual observation, chemical titration, and instrumental measurements such as acidity meter and sugar meter. These methods are time‐consuming, laborious, and destructive, and they cannot be used to detect batch samples (Porep, Kammerer, & Carle, 2015). Due to these limitations, researchers pay more attention to discover nondestructive, easy and reliable detection methods based on optics, electromagnetics, acoustics, etc. (JiYong et al., 2012; Xu et al., 2017). Near‐infrared (NIR) spectroscopy is an emerging nondestructive technology to obtain the spectral information of the samples based on interactions between samples and light energy (Hu, Sun, Pu, & Pan, 2016). Compared with traditional methods, the NIR spectroscopy has the advantages of fast detection speed, no damage to the samples and high efficiency. As a matter of fact, NIR spectroscopy has been widely studied to test the quality of fruits and vegetables (Theanjumpol et al., 2019). NIR spectroscopy mainly includes two optical alternatives: “reflectance” and “transmittance.” Most of the current researches used the NIR reflectance spectra to establish prediction models for fruits and vegetables quality. Even though the reflection spectra can be used to establish a closely related prediction model, it was only reflected the local component content, not the quality indicators of the whole samples. However, the spectral information of the whole sample can be obtained using full transmittance near‐infrared spectroscopy, which can be used for the evaluation of quality of the whole sample.

Even though NIR spectroscopy has been proved to be feasible to detect the quality of fruits and vegetables, its practical application still has limitations. The spectra developed for analysis are sensitive to variations in temperature, and calibration transfer problems still existed in practical applications of NIR (Sheng, Cheng, Li, Ali, Agyekum, & Chen, 2019; Xu, Mo, Xie, & Ying, 2019). Most of the previous studies were conducted under laboratory conditions, and the temperature were kept constant (Suchanek, Kordulska, Olejniczak, Figiel, & Turek, 2017). However, in practical application, the temperature of refrigerated fruits and vegetables differs greatly from detection temperature. Fruits and vegetables have good light transmittance due to high moisture content. In the photoelectric signal acquisition, temperature as an important disturbance factor changes the optical properties of fruits and vegetables and significantly affects the signal intensity, leading to wavelength shifts in absorbance response (Arendse, Fawole, Magwaza, & Opara, 2018). The detection of wines using NIR spectroscopy especially at the spectra region of 970–1,400 nm has been proved to be affected by the temperature, and the optimal temperature for testing was found to be 30–35°C (Cozzolino et al., 2007). In order to compensate for the influence of temperature on modeling, the mixed temperature correction method and partial least squares regression (PLSR) models for prediction of sugar content of molasses have been developed by combining spectral data at different temperature conditions (Chapanya, Ritthiruangdej, Mueangmontri, Pattamasuwan, & Vanichsriratana, 2018). For apple fruit, long‐term storage is required to meet the demands of annual supply. And the storage temperature is generally lower than 4°C, while the detection of apple quality is conducted under the room temperature, the significant temperature difference will lead to unstable detection results. Therefore, it is necessary to take measures to correct the temperature, reduce the influence of temperature on modeling, and improve the applicability of the model.

In addition to being influenced by external environment, NIR spectroscopy consists of overtones and combinations of infrared spectroscopy region, leading to overlapping of spectra (Saeys, Nguyen Do Trong, Van Beers, & Nicolaï, 2019). Moreover, a large number of spectral variables including irrelevant information need long data processing time and reduce the prediction accuracy, as a result, limiting its online applications. Recently, some variable selection methods, including synergy interval (SI) (Zhang, Xu, Wang, Tian, & Li, 2018), competitive adaptive reweighted sampling (CARS) (Guo, Wang, et al., 2019), ant colony optimization (ACO) (Yang et al., 2017), and uninformative variable elimination (UVE) (Li, Sun, & Cheng, 2016), have been studied to improve the stability and accuracy of modeling. CARS and SPA methods have been selected to determine the optimal wavelengths for prediction of apple SSC, and the prediction correlation coefficient (*R*_p_) up to .919 with prediction root mean square (RMSEP) of 0.592 (Fan et al., 2019). Hyperspectral imaging (HSI) coupled with wavelength selection algorithms such as CARS, SPA, and RF has also been developed to select effective wavelength and establish partial least squares (PLS) models for apple SSC prediction, and the obtained best results were *R*_p_, RMSEP values of 0.917, 0.453 °Brix (Zhang et al., 2019). Compared with full spectra models, variable selection method can effectively simplify the model and provide the basis for the practical application.

In this study, a new strategy was developed for detection storage quality of apples. Apple samples were stored at 4, 18, and 25°C, and NIR spectra were collected using portable NIR (590–1,250 nm) spectrometer. We established and compared the performance of quantitative detection models of apple SSC, VC, TA, and firmness under different temperatures and analyzed the effects of temperature on the models. The effects of temperature were compensated by using the mixed temperature correction (MTC) method and improved the performance of models. The optimal variables were determined by using variables selection methods such as UVE, CARS, UVE combined with SPA, and CARS combined with SPA, and the prediction performance of models was further improved based on the temperature compensation models. Consequently, NIR transmittance spectroscopy‐based feasible and reliable strategy was developed to detect postharvest quality of apples during storage.

## 2 MATERIALS AND METHODS

### 2.1 Apple samples

A total of 396 “Fuji” apples without any damage and with uniform shape, size, and maturity were purchased from local markets and rapidly delivered to the laboratory. In order to detect quality of apple samples at storage conditions with different temperatures. The apples were divided into three groups, and each of the group included 132 samples. Then, they were stored at three temperature conditions (4, 18, and 25°C), and the relative humidity was set as 80%, respectively.

### 2.2 Acquisition of NIR spectra

The NIR spectra were collected in transmittance mode by a portable NIR spectrometer USB 2000+ (Ocean Optics) in the region of 590–1,250 nm with the spectral resolution of 3 nm. This is a prototype inspection device, with a film‐coated flat convex lens, which has high transmittance to the detection band and low transmittance to the infrared band, thus avoiding the thermal damage of fruits. Every 2 days, five samples were taken out from each group to collect NIR spectra. For each sample, the NIR spectra were collected at the equatorial position with 120° rotation angle, resulted in three measured spectra, and the average value of the three spectra was taken as the final spectral data.

### 2.3 Reference data measurement

After the NIR measurement, the reference data of each apple sample including firmness, SSC, TA, and VC were obtained using standard methods (Feng, Zhang, Adhikari, & Guo, 2019). The detection steps are as follows: First, the firmness of each apple was recorded through physical property analyzer (Stable Micro Systems), the P/5 probe was selected, and the distance of puncture was 8 mm with the test speed of 1.5 mm/s (PérezMarín et al., 2019). Then, apple juice was taken to measure SSC, TA, and VC. The apple SSC was measured using digital refractometer (ATAGO). TA was measured according to acid–base titration method and calculated by the amount of sodium hydroxide consumed and expressed as mass percentage of citric acid. VC was evaluated using spectrophotometer (METASH) at 245 nm and calculated using standard curve. In order to reduce the influence of random errors, three tissue blocks from the equatorial position with 120° rotation angle of each sample were measured, and the average value was taken as the final quality parameter value.

### 2.4 Processing the spectral and reference data

#### 2.4.1 Spectra preprocessing

The original spectral data were converted into relative absorptivity (*A*) via equation: *A* = log (1/*T*) (Zhang, Wu, Zhang, Cheng, & Tan, 2017), in which *T* referred to transmissivity. Then, the spectral pretreatment methods including Savitzky–Golay (SG) (Guo, Li, et al., 2019) smoothing, standard normal variate (SNV) (Ma, Li, Inagaki, Yang, & Tsuchikawa, 2018), and multiplicative scatter correction (MSC) were used to remove the noise and baseline interference in spectral signals. The preprocessed spectra were used for further processing and establishing prediction models of apple quality (Wang & Xie, 2014).

#### 2.4.2 Temperature calibration

Considering the influence of temperature on the models, the mixed temperature correction (MTC) method was proposed to process the spectra. MTC method combined the spectral data of samples under different temperature conditions to establish a model, and the temperature information was involved in the model and analysis (Chapanya et al., 2018). In the processing of MTC, the accuracy of the prediction model depends on the number of representative samples of the calibration dataset, which needs to cover samples with a wide range of temperature changes. Therefore, the established model based on MTC contained the variation information of sample temperature, which enhanced the adaptability of the model to temperature changes.

### 2.5 Spectral variables selection

Near‐infrared spectroscopy contains a large number of spectral variables, some of which are irrelevant, redundant, and collinear information, hence causing the increase of data processing time and also interfering with the establishment of the model and affecting the stability and prediction accuracy of the model (Ouyang, Zhao, Pan, & Chen, 2016). Therefore, it is of great significance to select the effective variables using variable selection methods. The common variable selection methods include UVE, CARS, and SPA. Among them, the UVE and CARS could eliminate the variables with irrelevant information but the number of the retained variables is still very large. Therefore, SPA was usually necessary to further eliminate collinearity variables retained and reduce the number of modeling variables.

Uninformative variable elimination algorithm is based on the PLS regression coefficients, and it eliminates the wavelength variables without information and retains the effective variables by adding random noise variables to sample variables and interactive verification (Porep et al., 2015). CARS is an emerging variable selection method (Kutsanedzie et al., 2018; Wang et al., 2019). In the process of CARS, each wavelength is regarded as an independent individual, and the wavelength variables with larger absolute regression coefficients in PLS models are selected by adaptive reweighted sampling technology. At the same time, the wavelength variables with smaller absolute regression coefficients are removed. Finally, the key individuals of important information are to be retained, while the unimportant is removed. SPA is a forward variable selection algorithm, which minimizes the collinearity of vector space by extracting several characteristic wavelengths in the whole band and eliminating redundant information in the original spectral matrix (Fan et al., 2019; Guo et al., 2016).

### 2.6 Establishment and evaluation of models

Partial least squares is a quantitative analysis method frequently used for spectral analysis, which is insensitive to the spatial collinearity and large numbers of variables by projecting the predicted and observed variables into a new space (Huang, Lu, & Chen, 2018). In this study, PLS was first calibrated based on full spectra at different temperatures to evaluate the relationship between chemical and spectral data of apple such as SSC, TA, firmness and VC, and the effect of temperature on models. Then, spectra data processed by MTC were employed to establish PLS models and minimized the influences of temperature on model prediction. Finally, in order to further improve the prediction performance of the models, variable selection methods were used to determine efficient variables and establish PLS models.

To evaluate the prediction performance of the models, important parameters such as calibration correlation coefficient (*R*_c_) and *R*_p_ were used to obtain the degree of close correlation between variables, root mean square error of calibration (RMSEC) and RMSEP were used to measure the deviation between the observed value and the true value, and RPD was used to evaluate models prediction ability. The larger *R*_c_, *R*_p_ the lower RMSEC, RMSEP values showed the better models, and the RPD value is more than 2 shows that the model has potential practical application ability (He, Fu, Rao, & Fang, 2016).

(1) $$\text{RMSEC}=\sqrt{\frac{\sum_{i=1}^{n}{\left({\hat{y}}_{i}-y_{i}\right)}^{2}}{n}}$$

(2) $$\text{RMSEP}=\sqrt{\frac{\sum_{i=1}^{n_{\text{val}}}{\left({\hat{y}}_{i}-y_{i}\right)}^{2}}{n_{\text{val}}}}$$

(3) $$\text{RPD}=\frac{\mathrm{SD}}{\text{RMSEP}}$$

where *y_i_* is the practical value of apple quality indexes, while ${\hat{y}}_{i}$ is predictive estimate value. *n* is the sample number of correction set, *n*_val_ is the sample number of prediction set, and *SD* is the standard deviation.

## 3 RESULTS

### 3.1 Overview of spectra and statistics of reference data

**FIGURE 1:**
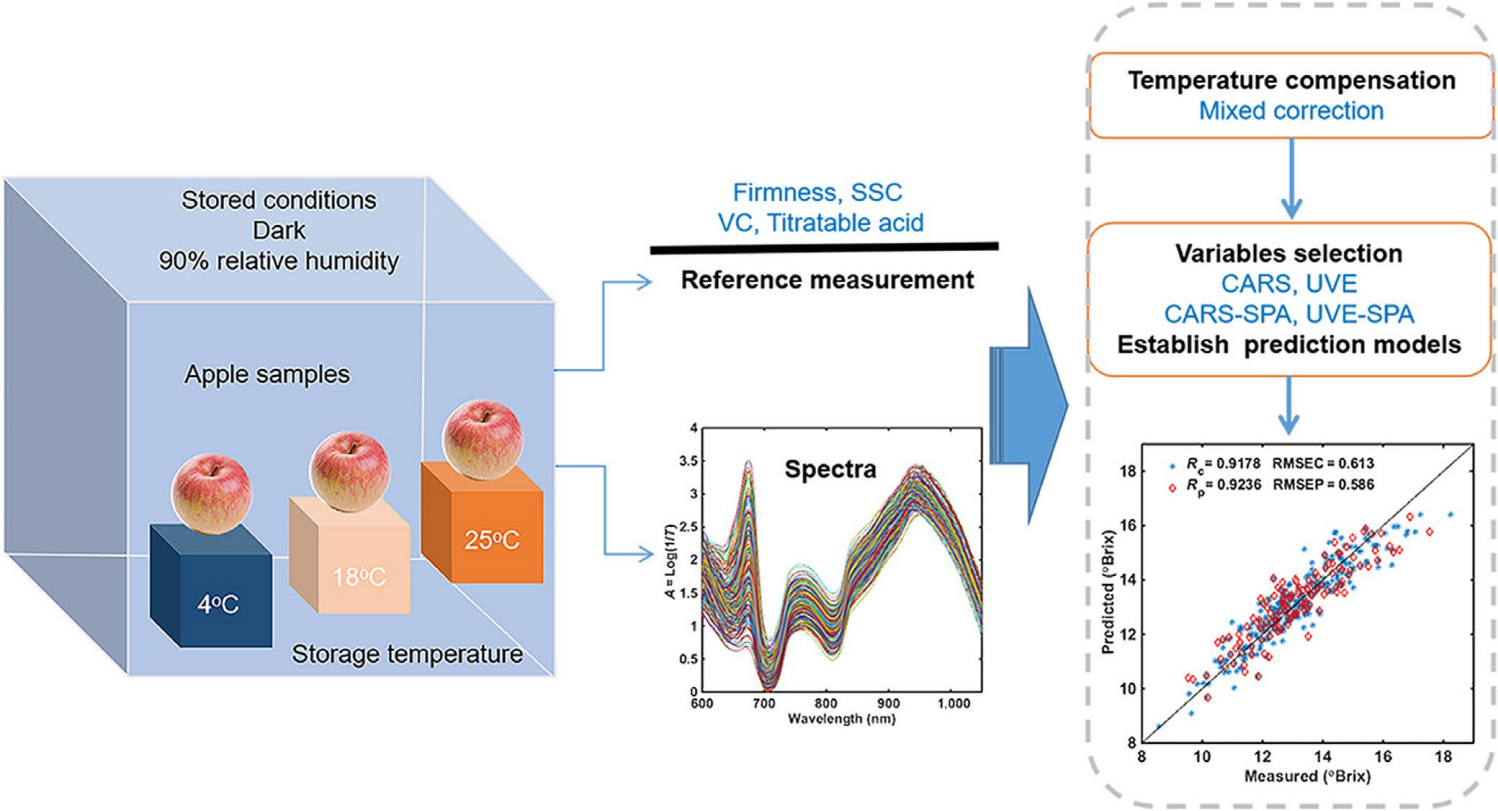
Schematic diagram of the experimental procedure. Near‐infrared transmittance spectroscopy of apple samples at different temperatures were collected, and a variety of variables selection methods were used to establish prediction models of main quality properties based on the reference measurements

Due to obvious edge noises in the front and the end of the spectral region, the wavelength range of 600–1,050 nm was selected for spectral analyzing and modeling. Figure 1 showed the representative transmittance spectra with wavelength region of 600–1,050 nm from all detected samples. It can be seen that NIR spectra were sensitive to apple tissue components, the spectra collected from all samples have shown similar trends, and some obvious spectra absorption peaks were at the wavelength of around 675, 760, and 945 nm. Among them, the absorption peak at around 675 nm might be related to chlorophyll and anthocyanins of apple peel (Sánchez, Entrenas, Torres, Vega, & PérezMarín, 2018). The absorption peak at 760 nm might be associated with C–H fourth overtone band, and the absorption trend at around 945 nm can be classified as O–H second overtone band of the internal components of apples tissue such as carbohydrates, minerals, and water content, which involved the molecular bonds C‐H and O‐H (Li et al., 2016).

Table 1 summarized the distribution of apple quality indicators including SSC, firmness, VC, and TA. A total of 396 apples were used for establishing independent component models and MTC models, and the samples were first divided into calibration set and prediction set. In the development of MTC models, for the calibration set, the mean values of SSC, firmness, VC, and TA were 13.11°Brix, 1.415 kg, 23.9 mg/100 g, and 2.13%, and the standard deviations were 1.541°Brix, 0.243 kg, 4.464 mg/100 g, and 0.267%, respectively. For the prediction set, the mean values of SSC, firmness, VC, and TA were 13.11°Brix, 1.441 kg, 23.9 mg/100 g, and 2.13%, and the standard deviations were 1.526°Brix, 0.204 kg, 3.861 mg/100 g, and 0.2663%, respectively. The results of *SD* show that the statistical values of other three quality indexes have the lower dispersion and higher stability, while VC statistical values have higher discreteness, but their distribution of all conforms to the normal distribution, which can be used for modeling and analysis. And it is also noteworthy that there is no significant difference of mean values between the calibration sets and prediction sets, and all the parameters values of prediction set were within the range of the calibration set, which is of great significant to ensure the prediction accuracy.

**TABLE 1:** Descriptive statistics of apple quality parameters including SSC (°Brix), firmness (kg), TA (%), and VC (mg/100 g)

Sample	Calibration set				Prediction set			
	Min	Max	Mean	*SD*	Min	Max	Mean	*SD*
SSC	8.563	18.24	13.11	1.541	9.543	17.54	13.11	1.526
Firmness	0.557	1.934	1.415	0.243	0.668	1.910	1.441	0.204
VC	12.46	37.54	23.90	4.464	13.94	34.23	24.24	3.861
TA	1.557	3.055	2.131	0.2679	1.557	2.829	2.131	0.2663

### 3.2 Spectral preprocessing based on PLS modeling

For improving the prediction accuracy of apple quality, the spectral data of 600–1,050 nm range were first pretreated by SG, SNV, and MSC, respectively, and the PLS models were established to evaluate the effectiveness of the pretreatment methods. The results showed that the stability of PLS models has not been improved after pretreatment. Therefore, the original spectrum was selected for further data processing and analyzing.

### 3.3 Comparison of the independent models and MTC models

**FIGURE 2:**
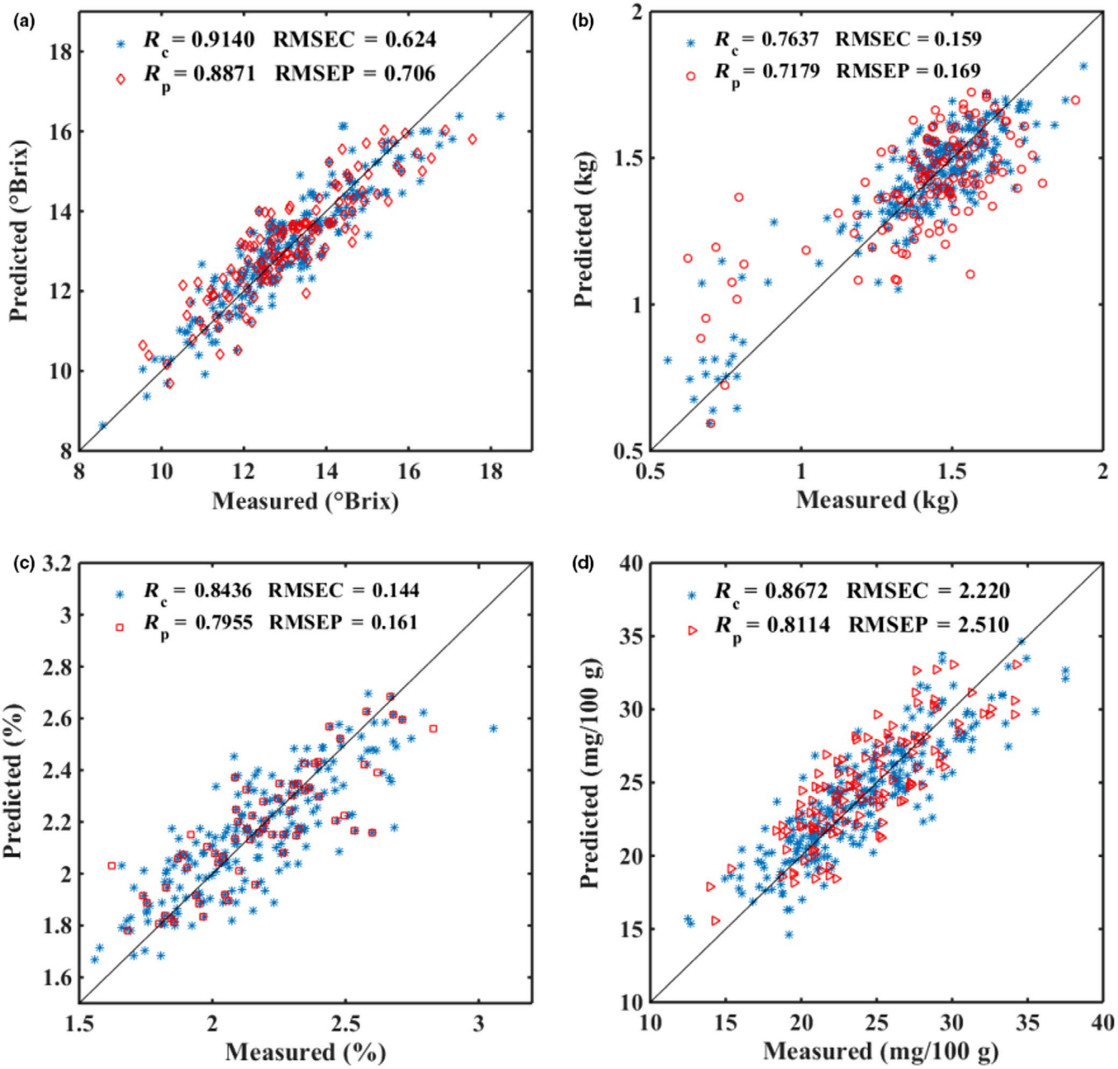
Scatter plots of calibration and prediction sets for apple SSC (a), firmness (b), TA (c) and VC (d), using mixed temperature compensation method during modeling

The PLS models for nondestructive detection of apple quality were established based on the different datasets at the wavelength range of 600–1,050 nm and in each apple quality index included three PLS‐independent models (4, 18, and 25°C) and two MTC models. These models were used for the analysis of the effect of temperatures on apple quality prediction, respectively. Figure 2a–d shows the scatter plots of prediction results of apple quality by PLS models. Table 2 shows the results of all models, and there is a slight difference between the results of different temperatures for the independent models, the optimal results of SSC, firmness, VC, and TA with *R*_P_ = .8678, RMSEP = 0.765 at 18°C, *R*_P_ = .7560, RMSEP = 0.107 at 18°C, *R*_P_ = .8089, RMSEP = 3.770 at 25°C, and *R*_P_ = .7763, RMSEP = 0.122 at 4°C, respectively. The prediction results of PLS models at 25 and 18°C were better than 4°C for SSC and firmness. Furthermore, the better prediction results were obtained when the temperature of the sample was consistent to that of the experimental environment. Compared the temperature compensation models with the independent models, the prediction accuracy of the models was significantly improved. In conclusion, the mixed temperature correction method significantly improved the prediction results of SSC, VC, and TA, and can be used to compensate the influence of temperature on the models. In this study, the optimal temperature compensation models for each quality index were further optimized using variable selection methods for effectively improving the prediction accuracy of models.

**TABLE 2:** The prediction results of SSC, firmness, TA, and VC in apple samples by independent component models established at different temperatures and mixed temperature compensation models

Quality parameters	Temperature(^o^C)	Calibration set		Prediction set		RPD
		*R*_c_	RMSEC	*R*_P_	RMSEP	
SSC	4	0.8243	0.921	0.8536	0.892	1.711
	18	0.8327	0.874	0.8678	0.765	1.995
	25	0.8309	0.767	0.8238	0.802	1.903
	MTC	0.9140	0.624	0.8871	0.706	2.161
Firmness	4	0.7861	0.152	0.7415	0.162	1.259
	18	0.7246	0.104	0.7560	0.107	1.907
	25	0.7208	0.148	0.7545	0.155	1.316
	MTC	0.7637	0.159	0.7179	0.169	1.207
VC	4	0.7561	3.500	0.7995	3.140	1.230
	18	0.7422	4.170	0.7714	3.730	1.035
	25	0.8077	3.730	0.8089	3.770	1.024
	MTC	0.8672	2.220	0.8114	2.510	1.538
TA	4	0.7726	0.130	0.7763	0.122	2.183
	18	0.8139	0.118	0.7536	0.144	1.849
	25	0.7317	0.134	0.7547	0.143	1.862
	MTC	0.8436	0.144	0.7955	0.161	1.654

### 3.4 Comparison of models established based on variables selection methods

#### 3.4.1 UVE‐PLS

**FIGURE 3:**
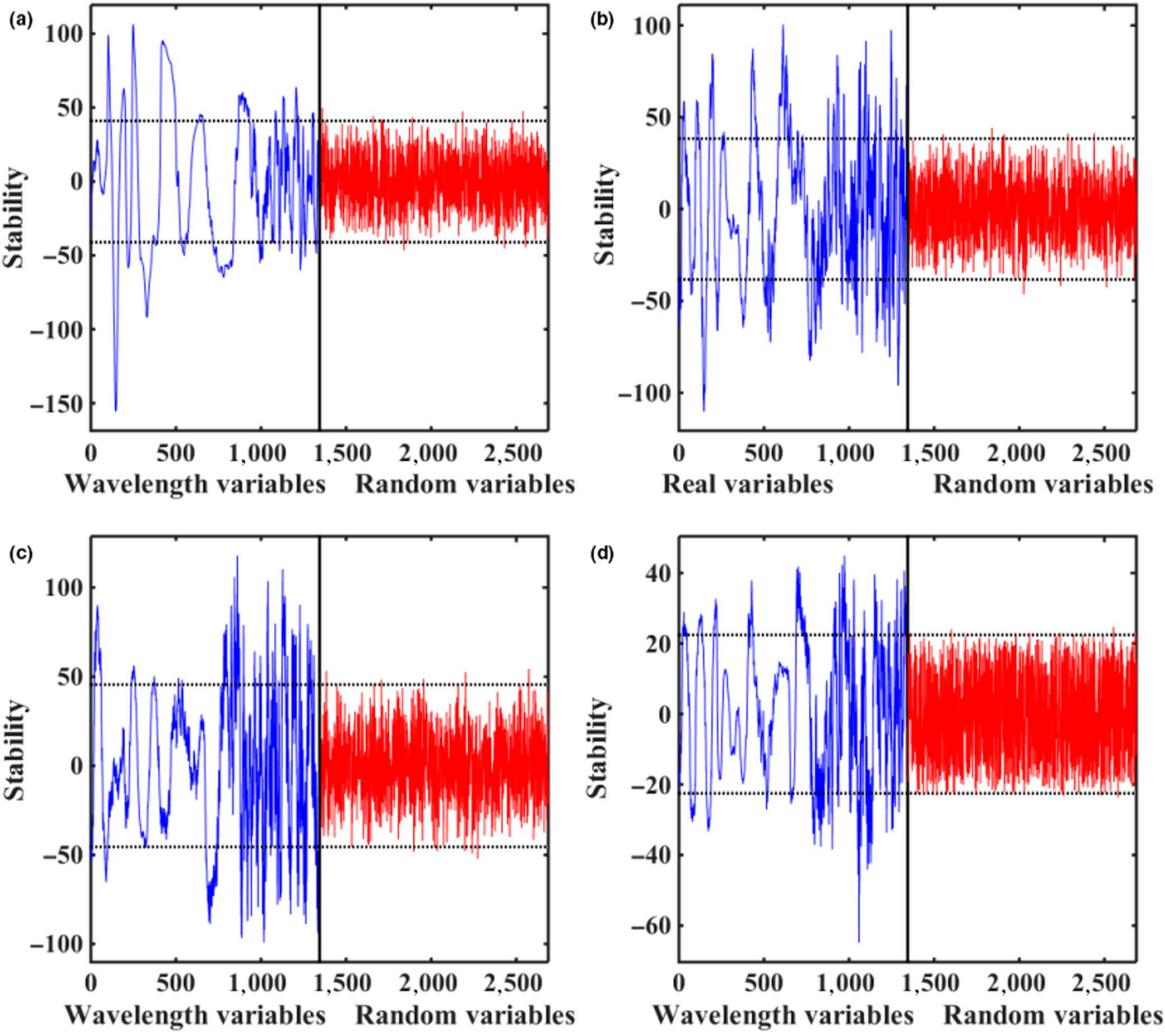
Characteristic variables selected by UVE for SSC prediction (a), firmness prediction (b), TA prediction (c) and VC prediction (d)

In the process of UVE, the maximum principal component number was set to 15, the random noise variables number was 1,344, and the stability value of random noise variables was 0.99, which was set as the threshold value. Figure 3a–d shows the stability of the UVE variable of apple quality detection. Due to the similar process of the UV selection algorithm of each quality index, this paper only describes the UVE variable screening process of SSC as an example. In Figure 3a, a total of 1,344 variables of blue line in the left region were real variables, while 1,344 variables of red line in the right region were added random noise variables. The two horizontal dotted lines above and below were the upper and lower thresholds of the stability of UVE variables. The variables between the two horizontal dotted lines were irrelevant variables, which were eliminated, while the variables outside the two horizontal dotted lines contained useful information and were retained. As a consequence, 610, 387, 342, and 523 spectral variables were retained for SSC, TA, VC, and firmness, respectively. Then, PLS models were developed based on the selected variables, and the results are shown in Table 3. Compared with the models based on the full spectra, the number of variables has been reduced by more than half. And the performance of models has been improved with *R*_p_ being .8983, .8633, .8293, and .7038 for SSC, TA, VC, and Firmness, respectively. The reason may be that some collinearity variable information has been removed by UVE.

**TABLE 3:** The prediction results of SSC, firmness, TA, and VC in apple samples by PLS models established using characteristic variables selected by different variable section methods

Quality parameters	Variable selection	Variable number	Calibration set		Prediction set		RPD
			*R*_c_	RMSEC	*R*_p_	RMSEP	
SSC	UVE	610	0.9124	0.630	0.8983	0.669	2.281
	CARS	83	0.9178	0.613	0.9236	0.586	2.604
	UVE‐SPA	49	0.8971	0.679	0.8902	0.696	2.193
	CARS‐SPA	32	0.9203	0.602	0.9007	0.668	2.284
TA	UVE	387	0.8817	0.126	0.8633	0.132	2.017
	CARS	108	0.8671	0.134	0.8684	0.133	2.002
	UVE‐SPA	108	0.8805	0.127	0.8579	0.133	2.002
	CARS‐SPA	54	0.8917	0.121	0.8606	0.136	1.958
VC	UVE	342	0.8713	2.190	0.8293	2.390	1.615
	CARS	83	0.8765	2.150	0.8922	1.850	2.087
	UVE‐SPA	31	0.8275	2.500	0.7832	2.580	1.497
	CARS‐SPA	40	0.8563	2.290	0.8288	2.330	1.657
Firmness	UVE	523	0.7956	0.147	0.7038	0.173	1.405
	CARS	94	0.8656	0.122	0.8207	0.117	1.992
	UVE‐SPA	77	0.7656	0.157	0.7325	0.162	1.548
	CARS‐SPA	59	0.8472	0.130	0.8179	0.115	1.869

#### 3.4.2 CARS‐PLS

**FIGURE 4:**
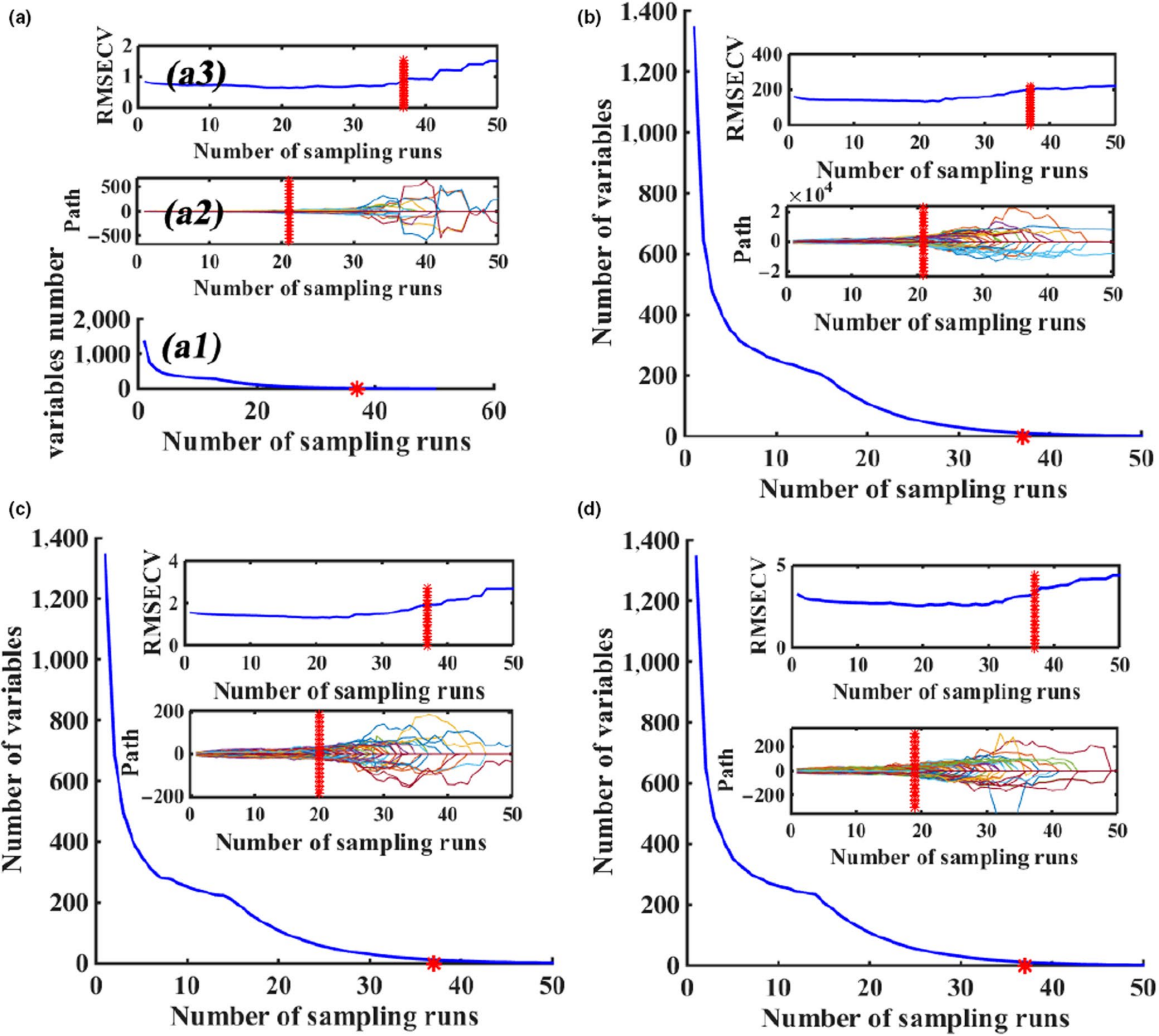
The process of CARS algorithm for SSC (a), firmness (b), TA (c) and VC (d)

In CARS algorithm processing, the sampling times of Monte Carlo were set to 50. Figure 4a–d shows the variable selection process for SSC, VC, TA, and firmness by CARS. It can be seen that, in the spectral range of 600–1,050 nm, the RMSEC values and the regression coefficient path of each wavelength changed with the increasing of sampling runs. In Figure 4a1, the selection speed of the wavelength variable changed from fast to slow, which reflected the process of selection from rough to fine. In Figure 4a2, RMSEC values descend first with the removal of uninformative variables, then gradually increased since some key variables were removed. And the optimum variable number was determined by minimum RMSEC value. Figure 4a3 shows the absolute coefficients at each sampling run for variables, and the variable of the lager absolute coefficient was more probable to be selected. As the result of the CARS calculation, 83 effective variables were selected for detection of SSC in apples. Similarly, 83, 108, and 94 variables were identified by CARS for VC, TA and firmness, respectively.

As shown in Table 3, compared with the full spectral PLS models, the prediction performance of models was all improved when using the selected variables by CARS algorithm. Meanwhile, the number of variables was reduced more than 90%, which showed that the CARS variable selection method removed irrelevant information and enhanced the signal‐to‐noise ratio of the model. When the variable selection effect of UVE and CARS was compared, the UVE improved the prediction effect of the model to a certain extent, while the variable selection ability of CARS was better than that of UVE, and the number of variables selected by CARS was lower than that selected by UVE. It might due to the variables retained by UVE still contained irrelevant information, which disturbed the prediction accuracy of the model.

#### 3.4.3 UVE‐SPA‐PLS and CARS‐SPA‐PLS

**FIGURE 5:**
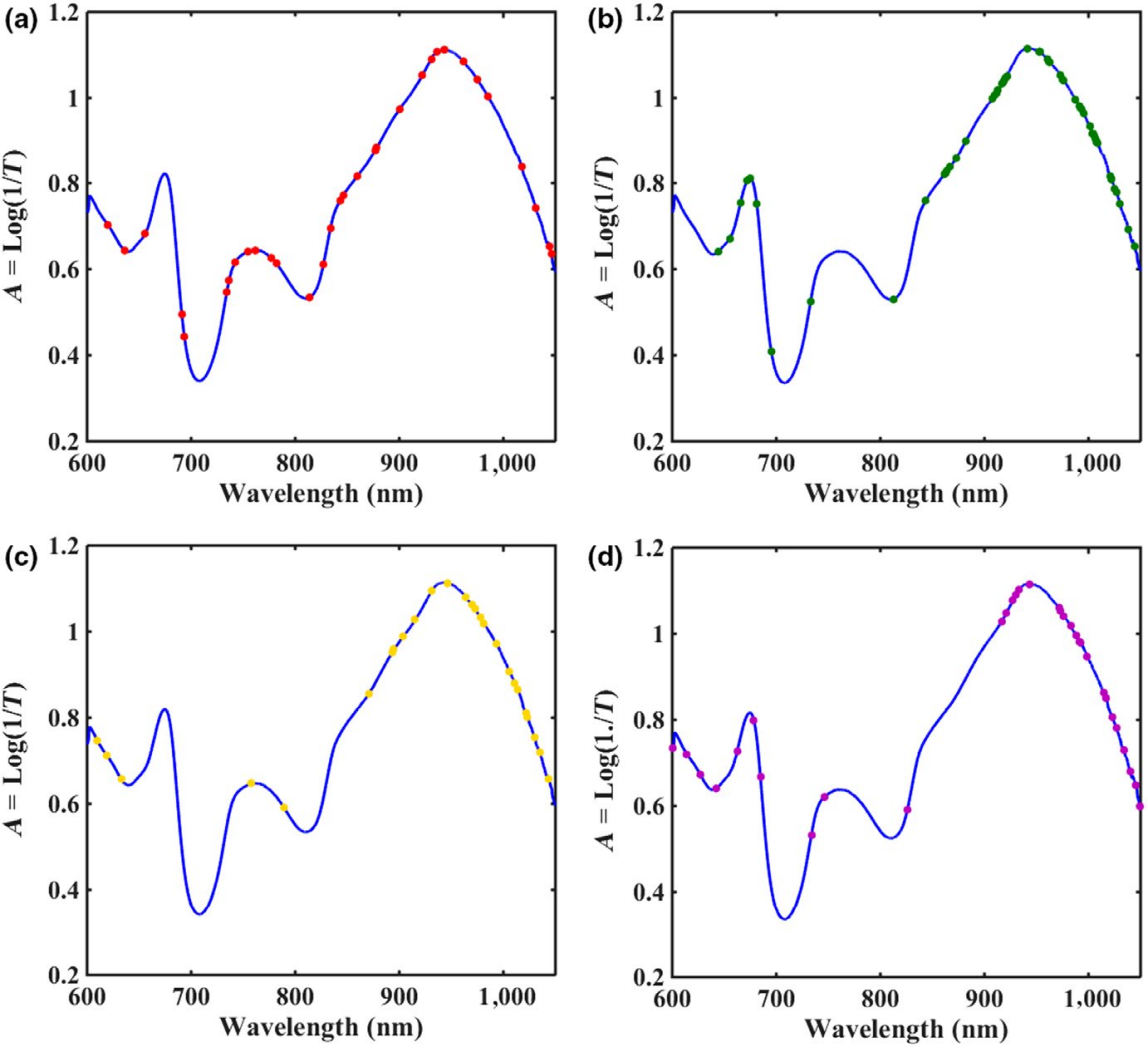
The distribution of characteristic variables determined by CARS‐SPA for SSC (a), firmness (b), TA (c) and VC (d)

Through the above UVE variable selection method, the number of variables used for quantitative analysis of apple quality was reduced by more than half from 1,344. However, some irrelevant variables might exist, which affected the stability of the models. In order to further simplify the model, SPA was developed to select characteristic variables based on the variables selected by UVE and CARS algorithms. Figure 4a–d showed the selected variables and corresponding wavelength points based on variables selected by CARS‐SPA. After CARS‐SPA processing, 32, 54, 40, and 59 characteristic variables were finally chosen for SSC, VC, TA, and firmness, respectively. And after UVE‐SPA processing, 49, 108, 31, and 77 characteristic variables were finally chosen for SSC, VC, TA, and firmness, respectively (Figure 5). As shown in Figure 4a–d, the selected characteristic wavelength points were mostly at the range of 600–700 and 900–1,000 nm, and the selection of wavelength points was slightly different for the four quality indicators, which reflected differences between response spectra of different quality indicators. Table 3 listed the calibration and prediction results of UVE‐SPA‐PLS and CARS‐SPA‐PLS models for SSC, VC, TA, and firmness of apples. Comparing the results of UVE‐SPA‐PLS and CARS‐SPA‐PLS models with UVE‐PLS and CARS‐PLS models, SPA greatly reduced the variables number and simplified the models . However, the prediction performances of models were slightly worse than that of UVE‐PLS and CARS‐PLS. The reason might be that the SPA removed some key variables and reduced the prediction accuracy.

### 3.5 Comparison of models by different variables selection methods

**FIGURE 6:**
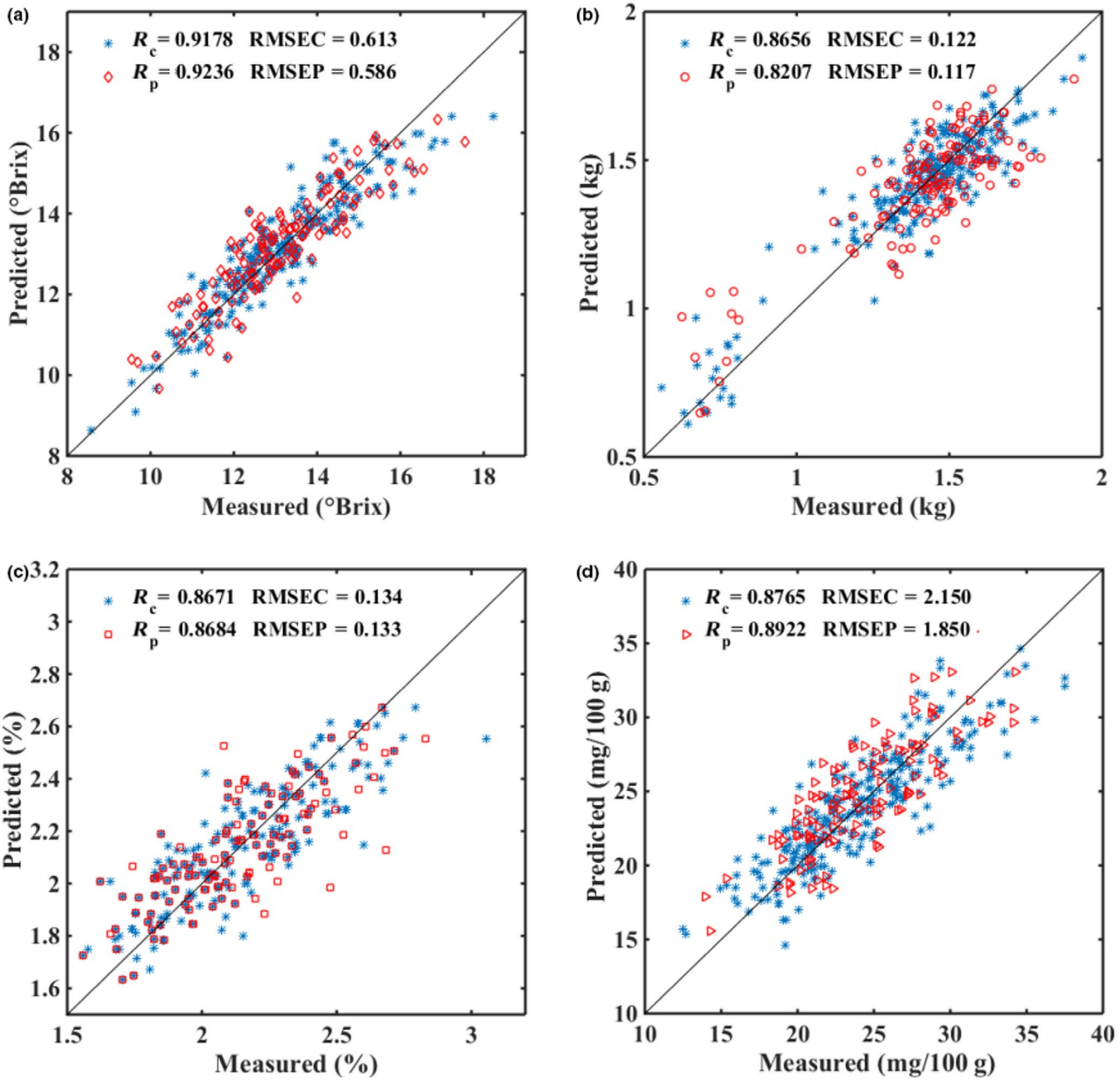
The prediction results of apple SSC (a), firmness (b), TA (c), and VC (d) by PLS models in calibration and prediction sets, of which the variables were selected by CARS

The full spectra data and characteristic variables determined by different variable selection methods were respectively developed to establish PLS models such as UVE‐PLS, CARS‐PLS, UVE‐SPA‐PLS, and CARS‐SPA‐PLS for quantitatively predicting quality indexes of apples. Table 3 listed the calibration and prediction results of all PLS models with full spectra and selected variables for SSC, VC, TA, and firmness of apples. And Figure 6a–d shows the scatter plots of the optimal prediction results of apple quality by CARS‐PLS models.

As a conclusion, the optimal results were obtained by CARS‐PLS models with *R*_P_ = 0.9236, RMSEP = 0.586 for SSC, *R*_P_ = 0.8684, RMSEP = 0.133 for TA, *R*_P_ = 0.8922, RMSEP = 1.850 for VC, and *R*_P_ = 0.8207, RMSEP = 0.117 for firmness, respectively. The results showed good prediction accuracy with RPD of 2.604 for SSC, approximate prediction accuracy with RPD of 2.087 for VC and 2.002 for TA, meanwhile, a poor prediction accuracy with RPD of 1.992 for firmness. Previous studies were mostly focused on the detection of apple SSC, and in this study, four quality indexes of apple were tested at the same time. For SSC and firmness, the result was slightly lower than previous studies (Fan et al., 2019; Ma et al., 2018; Ni, Zhu, Gu, & Hu, 2019), the reason may be that the distribution of SSC in apple was not uniform, other studies used the diffuse reflection method to obtain the accuracy SSC content of local apple, which cannot reflect the overall SSC content of apple, while the full transmittance spectrum can collect the spectral information of the whole apple SSC content in this study. For apple TA and VC, only few studies were existed, while this research detected a large number of apple samples and provided a potential of using near‐infrared transmittance spectroscopy to detect multiple quality indexes of apple. Furthermore, this study aimed at the quality detection of apple during storage, which shows the potential of NIR spectroscopy for apple storage quality monitoring.

## 4 CONCLUSION

This study revealed that the portable NIR spectroscopy system combined with a mixed temperature compensation method, and an appropriate variable selection method has the potential for the rapid detection of postharvest quality of apples. First, the NIR transmittance spectral data of apple stored at different temperatures were collected and used to establish models of apple quality, and the effects of temperature on the performance of models were compared. Then, the mixed temperature correction method was applied to reduce the effects of temperature on models. Based on which, the preprocessing methods such as SG, SNV, and MSC were used to improve signal‐to‐noise ratio of the models. Meanwhile, four variable selection methods including UVE, CARS, UVE‐SPA, and CARS‐SPA were employed to remove the variables, which were sensitive to temperature and improve the prediction performance of models. Results indicated that CARS‐PLS showed the optimal results with *R*_P_ = 0.9236, RMSEP = 0.586 for SSC, *R*_P_ = 0.8684, RMSEP = 1.330 for TA, *R*_P_ = 0.8922, RMSEP = 2.390 for VC, and *R*_P_ = 0.8207, RMSEP = 0.117 for firmness. These findings proved that NIR has the potential to be used in rapid detection of quality attributes of postharvest apple during storage.

## CONFLICT OF INTEREST

The authors declare that they do not have any conflict of interest.

## ETHICAL APPROVAL

This study does not involve any human or animal testing.

## INFORMED CONSENT

Written informed consent was obtained from all study participants.
